# Understanding the Behavioural Determinants of Nutrition Practices in Ultra-Endurance Runners in Ireland

**DOI:** 10.3390/sports14030109

**Published:** 2026-03-11

**Authors:** Tansy Ryan, Ed Daly, Lisa Ryan

**Affiliations:** Department of Sport, Exercise and Nutrition, School of Health, Sport Science and Nutrition, Atlantic Technological University, H91 T8NW Galway, Ireland; tansy.ryan@research.atu.ie (T.R.); ed.daly@atu.ie (E.D.)

**Keywords:** ultra-endurance running, sports nutrition, gastrointestinal symptoms, nutrition behaviours, COM-B model

## Abstract

Ultra-endurance running presents significant physiological demands, with adequate nutritional intake being critical for optimal preparation, performance, and recovery. However, athletes of this sport often consume an insufficient diet. Semi-structured interviews were conducted virtually with ten recreational ultra-endurance runners (age mean ± standard deviation 46 ± 12 years; eight male, two female), all with experience competing in ultra-endurance events, to explore their ultra-endurance experience, dietary intake, nutrition knowledge, and gastrointestinal symptom management. Interviews were transcribed verbatim and thematically analysed in accordance with the COM-B model and the Theoretical Domains Framework. Themes were defined under each of the three COM-B model components: Capability: (1) knowledge and skills, (2) the intention–behaviour challenge; Opportunity: (1) facilitators and barriers to optimal nutrition, (2) information sourcing and learning; and Motivation: (1) drivers of nutrition behaviours, (2) risk perceptions’ influence on fuelling strategies. Participants demonstrated strong psychological capability, that is, awareness of nutrition’s importance, yet limited behavioural regulation to translate this knowledge into practice. Nutrition strategies were largely self-directed, relying on peers and social media over professional support. Fear of gastrointestinal symptoms and time pressures further shaped dietary decisions. These findings emphasise the importance of evidence-based nutrition guidance to support performance and recovery while minimising gastrointestinal symptom risk. For practitioners and self-coached athletes, recommendations should reflect the practical realities and competing demands of ultra-endurance runners’ daily lives and be feasible within real-world settings.

## 1. Introduction

Ultra-endurance running, a term often used interchangeably with ultra-marathon running or ultra-running, is a form of endurance exercise defined by either distance or duration. The former describes an ultra-endurance run exceeding the well-known typical marathon length of 26.2 miles/42.2 km, while the latter defines an ultra-endurance run as a single bout of exercise that exceeds six hours in duration [1]. Events may be single- or multi-stage, with varying levels of self-sufficiency required. For example, in wholly self-sufficient events, participants must carry all required nutrition for the entire race, while semi-supported events offer intermittent refreshment points with food and hydration, and fully supported events provide frequent, organised nutritional provision. Additionally, the challenging nature of these events may be further exacerbated by rough terrain and harsh environmental conditions [2]. On a global scale, there has been a dynamic increase in ultra-endurance running event participation over the past 25 years, from approximately 34,401 total annual ultra-endurance running event participants in 1996 to 611,098 in 2018 [3,4].

Though serious adverse effects are uncommon, long-term health implications of ultra-endurance running include cardiovascular, respiratory, and musculoskeletal complications. For example, prolonged, high-volume running places significant stress on bones, muscles, joints, and connective tissues which, when coupled with inadequate nutrition, may contribute to osteoarthritic changes [5]. Additionally, gastrointestinal symptoms (GIS) are a prominent issue for ultra-endurance runners. These range from mild symptoms, such as belching and flatulence, to more severe discomfort, such as nausea and vomiting [6]. A range of intrinsic and extrinsic factors may exacerbate these. The former, intrinsic factors, include disorder or disease, internal bacterial composition (e.g., the gut microbiome), dietary intake, and nutrition strategies employed. Conversely, the latter, extrinsic factors, comprise variables such as exercise type, exercise duration, and surrounding environmental conditions [7,8,9].

Despite the significant importance of adequate nutrition and hydration practices for ultra-endurance athletes’ health and wellbeing, previous research has observed that this population often fails to meet intake recommendations [10,11,12]. In addition, a systematic review by Janiczak et al. [13] found athletes’ nutrition knowledge, a factor associated with their nutritional intake, to be low. The International Society of Sports Nutrition provides dietary recommendations and considerations for training and competing in ultra-endurance runs (see Table S1 in the Supplementary Material) [14]. Guidelines are defined as intake per hour, with energy, carbohydrates, and protein recommended at 150–400 kilocalories, 30–50 g, and 5–10 g, respectively. Regarding the optimal sourcing of these, athletes are advised to experiment with various foods and to take into consideration their individual food palatability, tolerances, and preferences. Though these recommendations exist, ultra-endurance athletes have been reported to favor other athletes’ experiences, opinions, and recommendations over those of qualified nutrition personnel, highlighting the influence of social opportunity and motivational factors on nutrition behaviours [15].

Little is known about the real-life experiences of ultra-endurance runners. Existing research investigating these athletes is often quantitative in nature, monitoring values such as blood biomarkers [11]. Previous reviews highlight the need to better understand food choice, nutrition knowledge, and diet quality using appropriate methods, as well as the challenges athletes face when translating recommendations into practice [16,17]. These gaps suggest potential deficits in the capability, opportunity, and motivation factors described in the COM-B model, yet no research has explicitly examined these mechanisms in ultra-endurance runners [18,19].

The COM-B model is a framework proposing three components—capability (C), opportunity (O), and motivation (M)—that are essential for a behaviour (B) to occur and can be used to guide intervention development (Figure S1 in the Supplementary Material). Capability refers to an individual’s physical and psychological capabilities to engage in a behaviour, such as their physical functional status and psychological skills and intentions. Opportunity encompasses their physical opportunities, such as available free time, and social opportunities, such as peer support. Lastly, motivation includes an individual’s automatic and reflective motivation. The former, automatic motivation, includes habits and emotional reactions, while the latter, reflective motivation, includes evaluation of experiences and future planning [20,21]. This model has been used to explore experiences, as well as barriers and facilitators, to physical activity and nutrition practices in both sport and non-sport populations [11,12,13,14]. However, this theory has yet to be incorporated into research on ultra-endurance runners [22,23,24].

Conversely, the Theoretical Domains Framework (TDF) is a theoretical framework that provides a lens through which to view the cognitive, affective, social, and environmental influences on behaviour. This framework consists of 33 theories of behaviour and behaviour change which have been clustered into 14 (originally 12) domains (see Table S2 in the Supplementary Material for examples of TDF domains and their constructs) [25,26]. Initially developed specifically to identify influences on health professional behaviour related to the implementation of evidence-based recommendations, the TDF was extended to explore other areas where behaviour change is important, such as physical activity engagement and nutrition practices [27,28].

Recognising the lack of theory-informed research in ultra-endurance running populations, the present study sought to gain an in-depth understanding of ultra-endurance runners’ lived experiences of nutrition behaviours and GIS management. Using the COM-B model and TDF, this study aimed to identify behavioural determinants, barriers, and enablers influencing the adoption of evidence-based nutrition practices.

## 2. Materials and Methods

### 2.1. Study Design

This research involved conducting individual interviews with ultra-endurance runners, with resulting transcripts thematically analysed in accordance with the Braun and Clarke [29,30] methodology for conducting qualitative investigations. Figure S2 in the Supplementary Material provides a visual overview of the research process.

### 2.2. Ethics

This project was granted ethical approval by the Research Sub-Committee of the Academic Council of Atlantic Technological University. An email outlining the nature of the research, eligibility criteria, and researchers’ contact details was initially distributed to relevant organisations and personnel. Individuals who contacted the researcher were sent a participant information sheet via email offering more details on the research, what it entailed, and the confidential nature of the data resulting from each interview. Consent was obtained from each participant prior to their partaking in a 1-1 digital interview with the researcher. All information was anonymised to protect the participants from identification.

### 2.3. Recruitment

Between January and June 2023, running organisations, clubs, and event organisers in Ireland were contacted and provided with study information and the author’s contact details. They were asked to share this information with their networks. A purposive sampling strategy was employed to target specific individuals with relevant ultra-endurance running experience. Snowball sampling was also used, whereby participants could recommend other athletes who met the inclusion criteria set for this investigation. These criteria were as follows: 18 years of age or older, any gender, healthy, living in Ireland, having any degree of running experience and having previously completed an ultra-endurance running event, considering themselves an “ultra-endurance runner”, having any history of GIS, and fluency in English. The exclusion criteria were as follows: diagnosed with a medical condition, pregnant or lactating currently or within the previous year, or having suffered an injury which halted the ability to run currently or within the previous year.

### 2.4. Data Collection and Analysis

The interviews were conducted virtually via Microsoft Teams and were semi-structured and iterative in their design, with questions based upon a previously developed and validated questionnaire by Scrivin et al. [31], which is freely available online as Supplementary Material to their publication. The lead researcher was integrated into a team with extensive experience in qualitative analysis, receiving training, participating in regular team discussions throughout the study, and keeping notes of research activities. Question responses were used to gather insight into nutrition’s role for ultra-endurance runners before, during, and after ultra-marathon events. Each participant was presented with questions regarding their experiences, which were broadly categorised into the following topics: (1) athlete history, (2) nutrition knowledge and sourcing, (3) the role of nutrition pre-, during- and post-ultra-endurance activity, and (4) GIS awareness and management. The audio recordings of each interview were transcribed verbatim and subsequently cross-referenced with the original audio to ensure accuracy. The resulting document consisted of n = 10 individuals’ interview transcripts (interview duration as mean ± standard deviation: 31 ± 11 min). The resulting data were reviewed and analysed in line with the Braun and Clarke recommended methodology for qualitative analysis, with themes subsequently mapped to the COM-B model and TDF to identify behavioural determinants, barriers, and enablers influencing nutrition practices [20,21,25,26]. This methodology comprised six key phases: (1) Familiarisation: transcriptions were read and re-read to become immersed and familiar with the data; (2) Initial Coding: succinct labels or “codes” were generated that captured and portrayed the critical information in the interviews; (3) Constructing Themes: the codes were examined and broader patterns were identified; (4) Reviewing and Refining Themes: the initial themes generated were checked against the coded data to ensure accurate reflection of information, with edits being made where necessary; (5) Defining and Naming Themes: each identified pattern or theme was given a name to represent it truly; and (6) Writing Results: the data extracts were woven together and linked to their most relevant theme, then discussed. The analysis was conducted from a realist/essentialist epistemological position, aiming to report the participants’ lived experiences as they described them. Coding was both inductive and deductive, with initial codes generated from the data, while subsequent organisation and interpretation were guided by the COM-B model and TDF domains. Themes were defined at a latent level, capturing underlying meanings and behaviours in participants’ accounts. A coding journal and theme development were developed by the first author, T.R., with these then discussed and agreed upon by the research team. The first author, T.R., conducted and transcribed each interview and analysed the results. The research team, E.D. and L.R., developed themes and sub-themes and subsequently reviewed them. Any disagreements were resolved through a research team group discussion.

### 2.5. Reflexivity and Data Management

The lead researcher’s background in sports nutrition and prior experience with ultra-endurance runners were acknowledged as both a strength, in terms of interpretative sensitivity, and a potential source of bias. Reflexivity was maintained through journaling, peer debriefing, and collaborative theme discussions. Coding was documented in a journal; themes were reviewed and refined collaboratively. Theory and investigator triangulation were employed in the present study. We acknowledge that member checking and systematic negative case analysis were not performed and highlight this as a limitation. Further questioning was carried out, when necessary, in order to draw out more information from the participants. Recruitment continued until data saturation was reached, defined as the point at which no new themes or sub-themes were identified from additional interviews.

## 3. Results

A total of n = 10 ultra-endurance runners participated (eight males, two females; age of those who disclosed: mean ± standard deviation 46 ± 12 years, range 29–79 years; Table 1). Participants varied in ultra-endurance experience), enabling exploration of nutrition behaviours through the COM-B framework and TDF.

Analysis of resulting interview transcripts resulted in the identification of six overarching themes, mapped to the components of the COM-B model and linked to their relevant domains of the TDF. These were: Capability: (1) knowledge and skills, (2) the intention-behaviour challenge; Opportunity: (1) facilitators and barriers to optimal nutrition, (2) information sourcing and learning; and Motivation: (1) drivers of nutrition behaviours, (2) risk perceptions’ influence on fuelling strategies (see Figure S3 in the Supplementary Material). Tables S3 and S4 in the Supplementary Material offer detailed contextual information on participants’ running backgrounds, nutrition knowledge, information sources, fuelling practices, gastrointestinal symptom management strategies, and commonly included or avoided foods, beverages, and supplements before and during ultra-endurance events.

### 3.1. Theme 1: Capability

The first component of the COM-B model, capability, refers to an individual’s capacity to engage in a behaviour. This encompasses both their physical abilities, such as fitness level, and psychological abilities, such as mental state and knowledge, to perform a behaviour and achieve the desired outcome. This aligns with the psychological capability component of the TDF, reflecting the athletes’ knowledge and skills necessary to enact effective nutrition behaviours. Two themes relating to participants’ capability became evident through analysing interview data. These were (1) nutrition knowledge and skills, and (2) the intention–behaviour challenge.

#### 3.1.1. Sub-Theme 1: Knowledge and Skills

Participants reported moderate self-perceived nutrition knowledge (scores 5–8/10, mean ~6.5), demonstrating awareness of energy, carbohydrate, fluid, and salt intake. Most participants reported eating and drinking ad hoc during training and events, guided by feelings of hunger and thirst. Knowledge of supplements and medication to support performance was also evident. Over half (n = 7) of participants included salt tablets in their event nutrition protocols; however, they were often ingested ad hoc, primarily dependent on when participants believed they were becoming dehydrated.

P6: “when you’re running, you get like white salt, kind of encrusting your face, and that’s a sign that you need to take on your salts as well. So these are things you learn”.

Many participants were aware of nutrition strategies that they could employ in the lead-up to an event, though many admitted they “tried to eat a lot of carbs……didn’t really have a proper approach” (P4) and would instead generally aim to eat “a couple of days before……be eating pastas and pizza’s and stuff like that” (P5). This demonstrates a gap between reflective motivation and procedural capability, whereby athletes understand what should be done but struggle to consistently implement structured strategies, relying instead on intuitive, experiential decision-making. Instead, they found themselves oftentimes “nibbling a little bit more” (P2) in the run-up to an event. Additionally, this time was often coupled with switching to leaner meats such as chicken and turkey, while simultaneously making intentional efforts to “avoid loads of sugar, biscuits, chocolate, sweets” (P4). The latter, in particular, was in an attempt to avoid GIS and feeling “a bit off ……iffy on a run” (P4).

During events, the majority (n = 9) of participants typically relied on high-sugar, fast-acting carbohydrate products including gels, flapjacks, jellies, and drinks. The exception to this, P3, avoids food before running, stating that he had been “doing it for years…and there’s no science to prove that I’m doing wrong” (P3). This participant considered himself “fairly knowledgeable” on nutrition, noting he has “enough confidence for [his social media account sharing nutrition information and recommendations]”.

P3: “I try not to eat about four hours before any runs. If it was a race I won’t eat at all before”.

For those who did fuel during events, eating and drinking periods were separated by timing, often approximately every 30 to 60 min, or distance, whereby food and fluid were ingested after specific distances. For example, one participant explained, “I would have practiced ok every 40 min. I have to take on some nutrition and then after every hour and a half I try and have something more substantial like uh, small bit of a sandwich” (P5).

One participant considered themselves extremely regimented with regard to planning their during-event nutrition and timing their food and fluid intake, noting he “had the spreadsheet with all my foods and with how many carbs, how many calories, what each portion would weigh, because I had to carry it in my bag” for the Marathon des Sables self-sufficient event (P10). However, this participant had a background in sport science with a previous career in sport.

In contrast, post-event nutrition was often undervalued, with many participants demonstrating limited knowledge of its importance for recovery. One participant, who originally began running as a weight-loss strategy, explained that they tried not to indulge after an event as “you don’t wanna be like piling on the weight afterwards” (P6). Here, the focus was on weight management rather than optimising nutrient intake for recovery. This suggests that although athletes may be aware of fuelling strategies for supporting event preparation, performance, and recovery, knowledge of specific evidence-based protocols and their reasoning is often lacking, leaving participants at risk of inadequate nutrition intake.

An outlier within the sample was one participant who, after discussions with a friend who was a sports nutritionist, avoided eating at all prior to and during events, a strategy they termed “running on empty” (P3). They believed that “food is sometimes poison, even the good food”, and that carrying food and liquids would expend energy better used to enhance performance, as it would make them lighter, less likely to experience GIS, and ultimately faster in events. Occasionally, this individual would bring sachets of baby food, consisting of pureed fruit, on long mountainous runs, but primarily relied on meditative breathing and touching their natural surroundings for energy.

P3: “Breathing the air, touching the trees, you know, cause at the end of the day, trees give us energy, you know?”.

This self-confessed “adrenaline junkie” described themselves as “always wanting more and more”, a behaviour that was mirrored in their nutrition practices. P3 described pushing their limits with extreme fasting: “I decided to push the limit…12 days nonstop. I was still training too”. While this represents an unusual outlier, it illustrates the broader pattern of ultra-endurance runners experimenting with extreme strategies. This lasted for 12 days and resulted in them feeling “like a new person”, with more energy, better vision (as they usually need glasses), and mental clarity.

Together, these accounts highlight that while participants possessed a general awareness of nutrition principles, this knowledge was often experiential rather than evidence-based, suggesting these athletes would benefit from clear, evidence-informed guidance tailored to the unique demands of their sport. Specifically, these findings reflect deficits in the TDF domains of knowledge and procedural skills, highlighting the need for interventions targeting both understanding and application.

#### 3.1.2. Sub-Theme 2: The Intention–Behaviour Challenge

When describing their nutrition intake across an ultra-endurance running season, participants highlighted a clear contrast between their pre- and during-event practices and their post-event behaviours. During the fortnight leading up to an event, participants became mindful of their nutritional intake and hydration status. They began making intentional efforts to reduce what they termed “junk foods” or “stodgy foods”. Carbohydrate loading was a commonly mentioned strategy, although participants frequently reported inconsistent implementation, with some experiencing nausea or discomfort during this phase. One athlete noted,

P3: “You’d be cutting back on your carbs or something in the run up to the event and then you’d load up with carbs, but that didn’t really work all that well for me. Remember I used to feel bit sick about it all”.

Carbohydrates remained the primary fuel source during races, most often consumed in the form of high-sugar products. In both instances, these strategies were rarely monitored or structured, leaving athletes at risk of ineffectively carbohydrate loading before events and failing to maintain adequate fuelling or hydration during competition. This highlights a clear gap between athletes’ knowledge and their consistent application of evidence-based nutrition strategies. Within the COM-B, this represents a capability–motivation interaction, whereby reflective motivation (intentions) is insufficient to drive consistent behaviours, illustrating challenges in behavioural regulation. This gap became even more evident in discussions relating to the post-race, or recovery, period, which was seldom a focal point in participants’ nutrition approaches. The post-race period was typically described as comprising foods and beverages avoided in the lead-up to competition. This “cheat week” was a time for athletes to “do what I want” (P8), usually involving indulging in alcoholic beverages, takeaways, and foods like crisps, chocolate, and cake.

This period was often accompanied by a sense of relief and freedom, where “the shackles are off” (P2). Only one participant described paying particular attention to their recovery in a regimented manner, noting, “Yeah, I always have some kind of a recovery shake” (P10). For the most part, participants ate and drank ad hoc during recovery, with many describing significantly elevated appetites where they could “literally just eat anything” (P4) following such a physiologically taxing activity.

One participant, who avoided eating during events, explained that their training continued as usual, viewing the event as an extended training run rather than a particularly demanding race. This general lack of focus on the recovery period suggests a gap in awareness regarding its importance. Participants approached this time with a sense of freedom from structured nutrition practices, leaving them at risk of consuming inadequate nutrients essential for optimal recovery, namely protein.

These findings highlight a need for targeted education interventions to inform ultra-endurance runners about the significance of the recovery phase and how best to approach it nutritionally. This aligns with the TDF domains of behavioural regulation and knowledge, suggesting interventions should focus on procedural skills and reflective planning for post-event nutrition.

### 3.2. Theme 2: Opportunity

The second component of the COM-B model, opportunity, refers to external physical and social factors that enable or hinder behaviour. Physical opportunity includes access to time, resources, and supportive environments, while social opportunity reflects cultural norms and interpersonal influences. Two opportunity-related themes emerged: (1) facilitators and barriers to optimal nutrition and (2) information sourcing and learning, illustrating how contextual factors shape ultra-endurance runners’ ability to adopt evidence-based nutrition practices.

#### 3.2.1. Sub-Theme 1: Facilitators and Barriers to Optimal Nutrition

Participants identified several physical and social opportunity factors that influenced their nutrition practices across training, competition, and recovery.

Facilitators included the accessibility of local events, running clubs, and online platforms, which enabled athletes to share experiential knowledge and practical advice. Peer interactions were particularly influential, with participants noting that “it’s still worth trying what other people [have tried]” (P1) and that “a lot of it is talking to other people who have done the distances before” (P2). Together, these factors highlight how social environments both support and shape nutrition behaviours within the ultra-endurance running community. Events often incorporated regular aid stations throughout their courses, usually comprising water and fruits. Though many participants brought their own nutrition to events, these aid stations acted as an important safety net for individuals to “dip into what [the race organisers] provide” (P1) when needed. One participant, P10, recounted how physical facilitators also included access to a Bikram yoga studio during his and his peers’ preparation for the Marathon des Sables ultra-marathon. This participant described how they “did a lot of running in a Bikram yoga studio” by running in circles in an approximately 10 × 5 m room while carrying the backpacks they would use in the event. This resulted in no heat-related difficulties for any of these athletes during the Sahara race, which reached temperatures of 47 °C that year.

Conversely, time constraints, work obligations, and family commitments hindered participants’ nutrition intake. For example, P1 discussed how they “tried to get [their] runs where they wouldn’t interfere with family time”, while P2 described how they would “Come home from work straight out training. So skip dinner until they (their children) get home”. Such commitments also limited participation in multi-day events abroad, with P10 noting, “You’ll be away from home for too long”. These constraints often led to inadequate meal planning and fuelling. P1 recalled, “I didn’t get to do my batch cooking, so I didn’t really have anything prepared or ready or much shopping in. So the last week I ended up eating a load of rubbish”, while P6 described resorting to fast food before a race: “the night before the last race… I had chips”. Ultra-endurance running, and activity in general, can result in substantial energy deficits. Failing to account for these in daily nutrition intakes previously led one participant to continuously find themselves binge eating at later points in the day: “I’d just sit there and snack on whatever I could find in the cupboards” (P4). The hindering nature of these commitments extended to participants’ broader ultra-endurance endeavours. Two individuals explained how, though they would like to partake in more events, particularly extreme multi-day events in other countries, such as the Everest Base Camp (60 km), “You’ll be away from home for too long” (P10).

These examples reflect the TDF domain of environmental context and resources, demonstrating how athletes’ behaviour is dependent on practical constraints beyond personal knowledge or motivation. In recognising this, practitioners must ensure their recommendations recognise this need for quick, accessible, nutritious foods, and emphasise the importance of regular intake throughout the day to reduce the risk of disordered eating behaviours, such as binge eating.

#### 3.2.2. Sub-Theme 2: Information Sourcing and Learning

Most participants (n = 7) relied on trial-and-error methods to determine which nutrition strategies best supported performance. The advice and opinions of others within the ultra-endurance running community also heavily influenced the specific strategies participants adopted. P5 explained they looked to “other athletes, online, and tips…I’ve picked up off other racers…over the runs I’ve done and stuff myself as well, like I’ve tried stuff out myself” (P5) while P4 noted using “Google, and specifically trying to sort of tailor including Googling things that are relevant to runners or strength training”.

Notably, it became evident that participants were not actively seeking advice from nutrition professionals. One participant had discussions with regular access qualified professionals, as they had a previous sporting career and were now a qualified sport scientist. Another participant reported informal interaction with a friend who was a qualified sports nutritionist, who advised protocols sometimes contrary to guidelines, such as avoiding food entirely before and during events. However, the exact qualifications behind their sport nutritionist title were not disclosed.

P3: “Ohh, just by experience, just by doing, just by working out myself and my… one of my friends was a nutritionist, sports nutritionist for a long time, so you know, helped get her help with a lot of advice also as well”.

These findings highlight gaps in the accessibility and perceived relevance of evidence-based nutrition guidance for ultra-endurance runners, suggesting interventions should target both opportunity (access to resources) and capability (knowledge application) across COM-B components.

### 3.3. Theme 3: Motivation

The third component of the COM-B model, motivation, refers to an individual’s internal processes that drive and direct behaviour. This encompasses automatic motivation, such as habits and emotional responses, and reflective motivation, including their evaluation of lived experiences and future planning. Two themes relating to motivation emerged in this research: (1) drivers of nutrition behaviours and (2) risk perceptions influencing fuelling strategies. Specifically, reflective motivation aligns with the TDF domains of goals, intentions, and beliefs about consequences, while automatic motivation maps onto emotion-driven responses.

#### 3.3.1. Sub-Theme 1: Drivers of Nutrition Behaviours

Most participants described a childhood, though key activities were team sports such as soccer, hurling, and childhood games. Interests in running emerged later, frequently during early career stages marked by sedentary work or lifestyle changes. P4 explained they “Finished university……Got a desk job that was, you know, stopped cycling to my part time job, stopped walking everywhere, got a car, put a little bit of weight on then thought I want to do something to shift the weight”. For some, initial motivation often stemmed from a desire to lose weight, or experiencing a “life is short, kind of moment” (P5). Others had similar moments of realisation, like health scares, prompting them to begin running.

P2: “Fell out of love with the cycling, fell in love with the drink…… Got a bit of what we’ll say, a health scare… sort of said right, we’ll need to do something. So I took up the running”.

Many began running in small groups, initially aiming to improve general fitness levels and potentially participating in short-distance running events, either as a way of raising money and awareness for charity, or as a personal challenge, with no initial desire to one day complete an ultra-marathon. As training demands increased, nutrition practices evolved, often guided by trial-and-error. Motivations such as weight control, health maintenance, adrenaline seeking, and personal challenges continued to shape fuelling and recovery behaviours, reflecting how early psychological drivers influence ongoing nutrition decisions.

This demonstrates how motivators inform current nutritional behaviours, reflecting the influence of automatic and reflective motivational processes on adherence to fuelling and recovery practices. Importantly, these findings suggest that the psychological drivers behind taking up the sport also underpin ongoing nutrition behaviours, influencing how athletes balance health, body image, and performance through food.

#### 3.3.2. Sub-Theme 2: Risk Perceptions’ Influence on Fuelling Strategies

There was a shared opinion between participants that the gastrointestinal implications of ultra-endurance running were as significant as its physical implications. P5 noted “Your stomach can finish the race as easy as your legs…it’s torture”. Ultra-endurance running was seen as a considerably different sport to marathon running, requiring unique nutrition strategies for GIS management. One participant with experience in shorter distance events described how “it doesn’t work for the ultras” (P10) in relation to using the same nutrition strategies in ultra-endurance running as they had previously used in marathon running. Participants shared experiences of themselves or others with severe GIS, having “seen the horror stories of people forgetting to eat, not drinking” (P2) or times when “my stomach went to pieces” (P5). Even of the participants that reported not regularly experiencing GIS, this generally was because they employed specific strategies to avoid them. These were strategies that, rather than being recommended by a health professional, were often identified by individuals through experimentation.

P6: “You have to have your own food and things that you’ve tried and tested, and you know it’s not gonna make your stomach sick”.

Some avoided food entirely, with P1 recalling, “Been on the toilet for so long…so I stopped eating”. Common triggers included caffeinated gels, carbonated drinks, and high-fat “stodgy” foods, leading athletes to prefer bland, easily digestible options: “Just very bland…going to be easy to digest…not gonna cause me any problems” (P8). Salt tablets were often preferred to water to avoid “swishies”, and some created personalised supplement mixes: “mixing up the concoction of (their) own” (P8).

In stark contrast to other participants who focused on ensuring a supportive nutrition intake before and throughout events, one participant reported that they avoided eating entirely in the days preceding and during an event and would instead sit in the sun, reconnecting with nature to fuel their activities. On occasions in the past when they did consume food pre-exercise, they described a subsequent feeling “sloggy and horrible and bloaty” (P3). Moreover, some athletes described taking over-the-counter medications such as Domperidone, which treats nausea and vomiting, or Loperamide, a medication to decrease the frequency of diarrhoea, as precautionary measures to avoid GIS before an event. However, these practices were self-directed and undertaken without medical or professional guidance, posing potential risks of inappropriate medication use and associated health complications.

P2: “two Imodium before the start of the race. And I swear about that. I tell everyone I know”.

P5: “you pick up tricks from people, like I bring motilium”.

Three participants also noted that ultra-endurance running, although oftentimes considered a “running competition”, was more accurately known as an “eating competition” among competitors. Even those with what they considered an “iron stomach” had previously experienced GIS, resulting in their active avoidance of multiple energy gels during events.

P10: “I have a do have a pretty iron stomach, you know, if you have too many, you know, they can make you feel a bit sick”.

It is evident from participant accounts that GIS are a prevalent concern within the ultra-endurance running community, and their management is considered critical for achieving optimal performance. Within COM-B, this reflects an interaction between automatic motivation (fear-avoidance) and reflective motivation (planned strategies), while TDF domains such as emotion and beliefs about consequences underpin these behaviours. For nutrition professionals, understanding the beliefs and risk perceptions that underpin athletes’ fuelling behaviours is essential to develop evidence-based, practical recommendations tailored to the unique physiological and psychological demands of ultra-endurance running.

## 4. Discussion

The present study used the COM-B model and TDF to explore ultra-endurance runners’ nutrition behaviours and GIS management. Nutrition behaviours were shaped by capability, opportunity, and motivation. Participants demonstrated sufficient psychological capability but often struggled with behavioural regulation, creating a gap between intention and action. Physical opportunities were limited by professional and family commitments, while social opportunities were shaped by a peer culture favouring informal advice. Automatic motivation, particularly anxiety surrounding gastrointestinal discomfort, frequently overpowered reflective planning. Together, these interacting elements explain why evidence-based recommendations were seldom implemented despite strong intentions.

Participants of this study considered their nutrition knowledge to be good, with self-rated nutrition knowledge ranging from 5 to 8 out of 10, consistent with previous studies in elite and amateur ultra-endurance runners [32,33]. This aligns with the psychological capability element of the COM-B model, and the ‘knowledge’, ‘skills’, and ‘memory, attention, and decision processes’ of the TDF, suggesting that these runners often possess the basic theoretical knowledge underlying the importance of nutrition for fuelling. Many relied on bodily cues, namely hunger, thirst, and the feeling of salt crusting on their skin, which reflects their intuitive approach to nutrition and hydration [34]. A minority of participants demonstrated advanced, structured approaches to nutrition for fuelling. Within the COM-B model, this reflects a breakdown in participants’ physical capability to translate nutrition knowledge into practice, and links to the ‘behavioural regulation’, ‘emotion’, and ‘reinforcement’ domains of the TDF, highlighting the need for interventions that translate theoretical knowledge into practical, evidence-informed strategies through applied fuelling workshops and personalised planning. This was particularly evident in the common but loosely implemented practice of carbohydrate loading [35,36]. Failing to meet the energy and, in particular, carbohydrate demands of ultra-endurance running is an issue that has been highlighted multiple times in previous investigations [11,37,38]. Similarly, the use of salt tablets to support hydration throughout events was commonly cited, but their ingestion was usually guided by cues of thirst and the sensation of salt crystals forming on the skin rather than evidence, indicating a misalignment between intention and scientifically supported protocols. Recommendations for sodium intake during ultra-endurance sports are highly individualised, depending heavily on sweat rates, which may be influenced by factors such as the racing environment, an individual’s clothing and anthropometric measurements, and training level [39]. The strong reliance on salt tablets by participants in this study is akin to previous study findings [40]. Opting for salt from tablets as opposed to food sources may be potentially to keep food volume low, a tactic P1 described employing for managing GIS, or a means to quickly obtain highly concentrated sodium into their system when experiencing salt crusting on their face like P6. However, this unstructured and unmonitored approach to sodium ingestion leaves ultra-endurance runners at risk of hypernatremia, whereby the sodium in the blood becomes abnormally high due to water loss exceeding sodium loss. This often presents similar to exercise-associated hyponatremia, in which the opposite situation occurs, leaving athletes at risk of mis-interpreting their needs and ultimately worsening their situation [41]. Viewed through COM-B, this reveals a disconnect between reflective motivation and physical capability; TDF domains involved include behavioural regulation, reinforcement, and beliefs about consequences. Interventions should therefore target structured fuelling plans, monitoring, and education on carbohydrate and sodium use.

Compared to pre- and during-event nutrition, post-event recovery received little attention. Participants described a relaxed approach, often indulging in takeaways, alcohol, and sweets with little regard for nutritional content. While research on ultra-endurance recovery nutrition is limited, Bonilla et al. [42] recommend a 4R framework—rehydration, refuelling, repairing, and resting. While rehydration needs vary depending on the athlete, event type and environment, a post-exercise rehydration strategy of 150% of the weight lost during exercise, alongside sodium, is the current general recommendation, while adequate carbohydrates and protein support glycogen replenishment and tissue repair. Few participants consciously monitored protein intake, suggesting recovery nutrition is undervalued. The indulgent and potentially suboptimal dietary choices during this time may potentially be rationalised by participants’ very high energy expenditure, which could make them feel justified in consuming foods that are convenient or enjoyable but not necessarily nutritious. Through COM-B, this reflects gaps in reflective motivation and psychological capability; TDF domains involved include ‘knowledge’, ‘beliefs about consequences’, and ‘reinforcement’. Interventions should therefore promote habit formation and education to normalise evidence-based recovery practices.

In this research, participants described being hindered by time constraints, as well as work and family commitments. In relation to the TDF, these findings link to the ‘environmental context and resources’ as well as ‘social influences’. Similar to Valentin et al. [43], who found training time was the main barrier regardless of gender, participants noted challenges balancing commitments with training. Comparably, social support from the ultra-endurance running community acted as a positive influence, with participants noting that they could rely on one another to work out problems and offer solutions. Viewed through a COM-B lens, these findings suggest interventions could improve physical opportunity through practical strategies like meal planning, while enhancing social opportunity via peer-led education supported by professionals.

Participants rarely cited nutrition professionals as a key source of information, instead relying on other athletes and the internet. This mirrors Mahoney et al. [19], who found health professionals were considered the least accessible or valued method of improving nutrition knowledge among runners. They instead placed more value on self-experimentation, trial-and-error, and the recommendations of other athletes. Ultra-endurance runners have been observed to be heavily committed to their community and often develop strong bonds with similar athletes, which may somewhat explain their tendency to refer to others for guidance [44,45]. Through the lens of the COM-B framework, these findings align with social opportunities and highlight that automatic motivation (such as emotional reinforcement and identity-driven factors) and reflective motivation (including health and self-improvement goals) work together to shape nutrition behaviours. Within the TDF, this aligns with the domains of ‘reinforcement’, ‘intention’, and ‘social or professional role and identity’. Interventions should therefore address both forms of motivation, encouraging balanced, evidence-based eating habits that support performance without undermining identity or enjoyment.

Participants often described health concerns or weight management as initial motivators for engaging in ultra-endurance running, which shaped their nutrition decisions. Some adopted the sport to control body weight, resulting in restrictive post-event eating, while others followed “clean eating” before races to feel prepared. This extends prior work showing health and self-fulfilment as motivators [46,47,48]. Within the COM-B model, this reflects the role of participants’ automatic motivation, where deeply ingrained habits, emotional responses, and health and weight concerns initially drove engagement in ultra-endurance running and associated nutrition behaviours. In terms of the TDF, this aligns with the ‘reinforcement’, ‘intention’, and ‘social/professional role and identity’ domains. On an automatic motivation level, fear of gastrointestinal distress led to strong avoidance habits such as skipping meals, restricting carbohydrate intake, or self-medicating, illustrating emotion-based regulation. These dual motivational forces influenced food choices more strongly than formal knowledge, helping explain why even well-informed athletes sometimes followed restrictive or inconsistent approaches. Behaviour-change strategies should therefore focus on reframing risk perception and easing GIS-related anxiety through gradual gut-training, psychological desensitisation, and education delivered by credible professionals.

The perception of risk, particularly relating to GIS, was a central driver of participants’ food choices. Although participants denied regularly experiencing GIS, avoidance of symptoms strongly influenced everyday dietary decisions. Consistent with previous research indicating that 65–96% of ultra-endurance runners experience GIS, particularly nausea and vomiting, participants described either having “seen the horror stories” (P2) or personally experiencing the consequences of improper fuelling [49,50]. Participants often avoided of energy gels, carbonated drinks, and “stodgy foods” (P8). This restrictive approach is widespread in ultra-endurance running, Zhao et al. [45] found that only 5.5% of runners reported no dietary restrictions. Although this approach can temporarily alleviate discomfort, over-restriction or fasting strategies pose significant risks., such as fatigue, impaired recovery, and conditions such as exercise-induced rhabdomyolysis, immunodepression, and Relative Energy Deficiency in Sport, known as REDs [49,51,52,53]. Moreover, a regular reliance on over-the-counter medications such as Loperamide or Domperidone to prevent GIS without prior consultation with a medical practitioner poses significant health risks, including an increased risk of cardiovascular events [50,54]. Within the COM-B model, this reflects the role of reflective motivation, where participants’ evaluation of risk of potential GIS regularly drives their food choices. In terms of the TDF, this aligns with the ‘knowledge’, ‘emotion’ and ‘beliefs about consequences’ domains, highlighting how ultra-endurance runners’ awareness of GIS risks, combined with their understanding of their impact on performance, shapes their nutrition strategies and food choices. These findings highlight the challenge ultra-endurance runners often face in balancing adequately fuelling for their sport while simultaneously avoiding and mitigating GIS, with management approaches rarely professionally informed. Practitioners supporting these athletes should recommend strategies which focus on evidence-based nutrition, hydration, and gut-training, adapted to each individual’s tolerance, instead of depending only on trial-and-error approaches [55].

Overall, these findings show that ultra-endurance runners’ nutrition behaviours are shaped by a complex interplay between capability, opportunity, and motivation. Although participants demonstrated strong theoretical knowledge and clear intentions, inconsistent application suggests gaps in procedural capability, limited physical opportunity, and the influence of automatic motivations such as anxiety around GIS. Interventions that address these interconnected factors by developing practical skills, creating supportive environments, and reducing emotion-driven barriers are likely to promote more effective and lasting behaviour change.

## 5. Limitations

The present study is not without limitations. Firstly, the sample size was small (n = 10). This is consistent with similar qualitative studies exploring ultra-endurance runners’ behaviours, where depth of data, rather than numerical generalisability, determines adequacy. Future research could recruit larger sample sizes to enable exploration of subgroup differences (e.g., gender, age, or performance level). The participants were recruited through advertising on social media and with running clubs, which may introduce the potential for selection bias as those with stronger opinions about nutrition may have been more inclined to participate. Additionally, individuals more active online may differ in nutrition knowledge from those less digitally engaged. Recruitment was conducted through running clubs, event-based dissemination, and social media, meaning the number of individuals who viewed the recruitment materials, declined participation, or were otherwise exposed but did not respond could not be quantified. This limits reporting of recruitment flow and response rates and should be considered when interpreting the findings. The reliance on self-reported data through interviews may be subject to recall bias and social desirability bias, potentially affecting the accuracy of the reported behaviours and practices. Future research could incorporate quantitative dietary information such as estimates of energy or macronutrient intake, as this would allow for more precise analysis of nutritional adequacy, better comparison with guidelines, and a clearer understanding of how dietary practices relate to performance and GIS. Future research should also ensure greater gender representation, as sociocultural and physiological factors may influence fuelling and recovery behaviours differently. Only two females participated in the present study, preventing meaningful analysis of gender differences. As well as this, future research could investigate why athletes lack confidence in health professionals and what drives their specific nutrition choices (such as carbohydrate loading and using salt tablets). Furthermore, this study focused primarily on athletes situated in Ireland, which may limit the cultural and geographical applicability of the findings. There were only two females included in this study, preventing meaningful analysis of gender differences. Future research should aim to include larger, more diverse sample populations to expand upon these findings. With larger cohorts, it may also be possible for researchers to stratify results by variables such as age and athletic level. Beyond these considerations, several methodological limitations should also be acknowledged in the current study. Although recruitment continued until no new themes emerged, there was no formal documentation of data saturation. Additionally, member checking with participants was not conducted to verify the accuracy of interpretations, and negative cases which may have challenged emerging themes were not systematically sought.

## 6. Practical Implications

The findings of this study highlight several practical implications for helping ultra-endurance runners optimise their nutrition practices and guide practitioners in supporting these athletes. Given the observed intention–behaviour gaps and reliance on experiential rather than evidence-based practices, interventions should move beyond general education toward concrete, behaviourally informed approaches. Behavioural strategies such as implementation intentions and self-monitoring tools can support athletes in planning and tracking nutrition behaviours consistently. Structured guidance from sport nutrition professionals can translate scientific recommendations into practical resources, including event-specific meal-planning templates, checklists, and short micro-learning modules covering carbohydrate intake, hydration, electrolyte management, recovery, and evidence-based GIS mitigation strategies. Gut-training protocols provide an avenue to gradually increase carbohydrate tolerance and reduce GIS risk, allowing athletes to test fuelling strategies in training and build confidence for competition. Embedding these resources within athlete communities, through running clubs, online forums, and race briefings, leverages social support while introducing credible expert input. Motivation can be enhanced through approaches such as motivational interviewing or digital self-monitoring, helping athletes reframe fears around GIS and reinforce adaptive behaviours, including consistent fuelling and structured post-event recovery. By aligning interventions with these behavioural mechanisms, practitioners can move beyond education alone and support sustained, context-appropriate behaviour change that enhances performance, promotes recovery, and reduces the risk of nutrition-related health complications. Additionally, future research should examine why ultra-endurance runners appear reluctant to engage with nutrition professionals, including perceived lack of sport-specific expertise, accessibility barriers, cost, or misalignment with athletes’ experiential and identity-driven approaches to learning. Understanding these factors may inform strategies to better integrate nutrition professionals within athlete communities in ways that enhance trust and relevance.

## 7. Conclusions

This qualitative study applied the COM-B model and TDF to explore ultra-endurance runners’ lived experiences, focusing on their nutrition behaviours, strategies for managing GIS, and the behavioural determinants, barriers, and enablers that shape the uptake of evidence-based nutrition practices. Themes were identified under each COM-B component: Capability: (1) knowledge and skills, (2) the intention–behaviour challenge; Opportunity: (1) facilitators and barriers to optimal nutrition, (2) information sourcing and learning; and Motivation: (1) drivers of nutrition behaviours, (2) risk perceptions influencing fuelling strategies. Participants demonstrated moderate knowledge and skills regarding nutrition principles yet struggled to translate this into structured fuelling and recovery practices. Time constraints often hindered nutrition planning and preparation, resulting in inadequate fuelling. Participants heavily relied on peer advice and trial-and-error approaches rather than the support of qualified professionals. Fear of gastrointestinal symptoms influenced fuelling practices, often leading to restrictive approaches due to their own, or awareness of others’, previous GIS experiences. Addressing these factors through practical, context-specific, and behaviourally informed support may help athletes optimise fuelling and recovery, minimise their risk of severe energy deficits, GIS, and other nutrition-related complications.

## Figures and Tables

**Table 1:** Participant characteristics (participants were given the choice not to disclose their age).

Participant Number	Gender	Age
1	Female	Not disclosed
2	Male	45
3	Male	49
4	Male	29
5	Male	39
6	Female	46
7	Male	39
8	Male	70
9	Male	Not disclosed
10	Male	54

## Data Availability

The original contributions presented in this study are included in the article/Supplementary Material. Further inquiries can be directed to the corresponding author.

## Supplementary Materials

The following supporting information can be downloaded at https://www.mdpi.com/article/10.3390/sports14030109/s1, Figure S1: A depiction of the COM-B model, its components capability (C), opportunity (O), and motivation (M), and examples of topics which exist within each sub-component, adapted from Michie et al. [20]; Figure S2. Illustration providing an overview of the research process and analytic steps undertaken, illustrating how data were generated, analysed, and interpreted within the COM-B and TDF frameworks; Figure S3. Behavioural determinants of nutrition practices in ultra-endurance runners who participated in the present study, mapped in accordance with the COM-B (capability, opportunity, and motivation) model of behaviour change and the Theoretical Domains Framework domains; Table S1: Table outlining the International Society of Sports Nutrition Position Statement recommendations for energy, carbohydrates, protein, fats, liquids, and sodium intake during single-stage ultra-endurance running training and racing, and how to mitigate and manage gastrointestinal symptoms (GIS) [14]; Table S2: Examples of The Theoretical Domains Framework (TDF, version 2) domains, definitions, and their comprising constructs, adapted from “A guide to using the Theoretical Domains Framework of behaviour change to investigate implementation problems” by Atkins et al. [56]; Table S3. Participants’ running history (when they started running, their initial reasoning for running, if they always had an interest in pursuing ultra-endurance running, and weekly training volume), whether they employ strategies to manage gastrointestinal symptoms (GIS), their self-reported level of nutrition knowledge rated on a scale from 1, no knowledge, to 10, extremely knowledgeable, and their sources of nutrition information; Table S4: Example foods and beverages that participants often focus on including or avoiding before and during an ultra-endurance running event, whether, when asked, they reported experiencing GIS, whether in their interview they described having experienced GIS, and the supplements they include in their diet.

## Abbreviations

The following abbreviations are used in this manuscript:

GIS	Gastrointestinal Symptoms
TDF	Theoretical Domains Framework

## References

[1] Scheer V., Basset P., Giovanelli N., Vernillo G., Millet G.P., Costa R.J.S. (2020). Defining Off-road Running: A Position Statement from the Ultra Sports Science Foundation. Int. J. Sports Med..

[2] Deutsche Ultramarathon Vereinigung e.V. https://www.ultra-marathon.org/.

[3] Thuany M., Gomes T.N., Villiger E., Weiss K., Scheer V., Nikolaidis P.T., Knechtle B. (2022). Trends in Participation, Sex Differences and Age of Peak Performance in TimeLimited- Ultramarathon Events: A Secular Analysis. Medicina.

[4] Ronto P. The State of Ultra Running 2020. https://runrepeat.com/state-of-ultra-running.

[5] Scheer V., Tiller N.B., Doutreleau S., Khodaee M., Knechtle B., Pasternak A., Rojas-Valverde D. (2022). Potential Long-Term Health Problems Associated with Ultra-Endurance Running: A Narrative Review. Sports Med..

[6] Hoogervorst D., van der Burg N., Versteegen J.J., Lambrechtse K.J., Redegeld M.I., Cornelissen L.A.J., Wardenaar F.C. (2019). Gastrointestinal complaints and correlations with self-reported macronutrient intake in independent groups of (ultra)marathon runners competing at different distances. Sports.

[7] Berger N.J.A., Best R., Best A.W., Lane A.M., Millet G.Y., Barwood M., Marcora S., Wilson P., Bearden S. (2024). Limits of ultra: Towards an interdisciplinary understanding of ultra-endurance running performance. Sports Med..

[8] Zhang L., Li H., Song Z., Liu Y., Zhang X. (2024). Dietary strategies to improve exercise performance by modulating the gut microbiota. Foods.

[9] Chantler S., Griffiths A., Matu J., Davison G., Jones B., Deighton K. (2021). The effects of exercise on indirect markers of gut damage and permeability: A systematic review and meta-analysis. Sports Med..

[10] Devrim-Lanpir A., Hill L., Knechtle B. (2021). Efficacy of Popular Diets Applied by Endurance Athletes on Sports Performance: Beneficial or Detrimental? A Narrative Review. Nutrients.

[11] Kinrade E.J., Galloway S.D.R. (2021). Dietary Observations of UltraEndurance- Runners in Preparation for and During a Continuous 24-h Event. Front. Physiol..

[12] Jiménez-Alfageme R., Álvarez J., Garbisu-Hualde A., Romero-García D., Giménez-Monzó D., Sospedra I., Ausó E., Martínez-Sanz J.M. (2024). Are the Dietary-Nutritional Recommendations Met? Analysis of Intake in Endurance Competitions. Nutrients.

[13] Janiczak A., Devlin B.L., Forsyth A., Trakman G.L. (2022). A Systematic Review Update of Athletes’ Nutrition Knowledge and Association with Dietary Intake. Br. J. Nutr..

[14] Tiller N.B., Roberts J.D., Beasley L., Chapman S., Pinto J.M., Smith L., Wiffin M., Russell M., Sparks S.A., Duckworth L. (2019). International Society of Sports Nutrition Position Stand: Nutritional Considerations for Single-Stage Ultra-Marathon Training and Racing. J. Int. Soc. Sports Nutr..

[15] Blennerhassett C., McNaughton L.R., Cronin L., Sparks S.A. (2019). Development and Implementation of a Nutrition Knowledge Questionnaire for Ultraendurance Athletes. Int. J. Sport Nutr. Exerc. Metab..

[16] Pelly F.E., Thurecht R.L., Slater G. (2022). Determinants of Food Choice in Athletes: A Systematic Scoping Review. Sports Med. Open.

[17] Blennerhassett C., McNaughton L.R., Sparks S.A. (2019). Factors Influencing Ultra-Endurance Athletes Food Choices: An Adapted Food Choice Questionnaire. Res. Sports Med..

[18] Lavoué C., Siracusa J., Chalchat É., Bourrilhon C., Charlot K. (2020). Analysis of Food and Fluid Intake in Elite Ultra-Endurance Runners during a 24-h World Championship. J. Int. Soc. Sports Nutr..

[19] Mahoney S.E., Wójcicki T., Carnes A.J. (2020). Sources of Nutrition Information in Recreational Ultra-Marathon Runners: A Mixed Methods Analysis. J. Hum. Perform. Extreme Environ..

[20] Michie S., van Stralen M.M., West R. (2011). The Behaviour Change Wheel: A New Method for Characterising and Designing Behaviour Change Interventions. Implement. Sci..

[21] Michie S. (2014). Implementation Science: Understanding Behaviour Change and Maintenance. BMC Health Serv. Res..

[22] Harman B., Kosirnik C., Antonini Philippe R. (2019). From Social Interactions to Interpersonal Relationships: Influences on Ultra-Runners’ Race Experience. PLoS ONE.

[23] Watkins L., Wilson M., Buscombe R. (2022). Examining the Diversity of Ultra-Running Motivations and Experiences: A Reversal Theory Perspective. Psychol. Sport Exerc..

[24] Bill T., Dessart G., Antonini Philippe R. (2024). Does Ultra-Endurance Passion Make Athletes Happy?. Sports.

[25] Michie S., Johnston M., Abraham C., Lawton R., Parker D., Walker A., “Psychological Theory” Group (2005). Making Psychological Theory Useful for Implementing Evidence Based Practice: A Consensus Approach. Qual. Saf. Health Care.

[26] Cane J., O’Connor D., Michie S. (2012). Validation of the Theoretical Domains Framework for Use in Behaviour Change and Implementation Research. Implement. Sci..

[27] Ndupu L.B., Staples V., Lipka S., Faghy M., Bessadet N., Bussell C. (2023). Application of Theoretical Domains Framework to Explore the Enablers and Barriers to Physical Activity Among University Staff and Students: A Qualitative Study. BMC Public Health.

[28] Carey S., Hogan S. (2025). Using the Theoretical Domains Framework and Behavior Change Wheel Framework Within the World of Nutrition Support. Nutr. Clin. Pract..

[29] Braun V., Clarke V., Weate P. (2016). Using Thematic Analysis in Sport and Exercise Research. Routledge Handbook of Qualitative Research in Sport and Exercise.

[30] Braun V., Clarke V. (2006). Using Thematic Analysis in Psychology. Qual. Res. Psychol..

[31] Scrivin R., Costa R.J.S., Pelly F., Lis D., Slater G. (2023). Carbohydrate Knowledge, Beliefs, and Intended Practices of Endurance Athletes Who Report Exercise-Associated Gastrointestinal Symptoms. Front. Nutr..

[32] Citarella R., Itani L., Intini V., Zucchinali G., Scevaroli S., Kreidieh D., Tannir H., El Masri D., El Ghoch M. (2019). Nutritional Knowledge and Dietary Practice in Elite 24-Hour Ultramarathon Runners: A Brief Report. Sports.

[33] Kosendiak A., Król M., Ligocka M., Kepinska M. (2023). Eating Habits and Nutritional Knowledge Among Amateur Ultrarunners. Front. Nutr..

[34] Costa R.J., Teixeira A., Rama L., Swancott A.J., Hardy L.D., Lee B., Camões-Costa V., Gill S., Waterman J.P., Freeth E.C. (2013). Water and Sodium Intake Habits and Status of Ultra-Endurance Runners During a Multi-Stage Ultra-Marathon Conducted in a Hot Ambient Environment: An Observational Field Based Study. Nutr. J..

[35] Burke L.M., Castell L.M., Casa D.J., Close G.L., Costa R.J.S., Desbrow B., Halson S.L., Lis D.M., Melin A.K., Peeling P. (2019). International Association of Athletics Federations Consensus Statement 2019: Nutrition for Athletics. Int. J. Sport Nutr. Exerc. Metab..

[36] Rothschild J.A., Kilding A.E., Plews D.J. (2020). What Should I Eat Before Exercise? Pre-Exercise Nutrition and the Response to Endurance Exercise: Current Prospective and Future Directions. Nutrients.

[37] Mahon E., Hackett A., Stott T., George K., Davies I. (2014). Macronutrient Consumption Prior to, and During, a Mountain Marathon. Am. J. Sports Sci..

[38] Hough P.A., Earle J. (2017). Energy Balance During a Self-Sufficient, Multistage Ultramarathon. J. Hum. Perform. Extreme Environ..

[39] Veniamakis E., Kaplanis G., Voulgaris P., Nikolaidis P.T. (2022). Effects of Sodium Intake on Health and Performance in Endurance and Ultra-Endurance Sports. Int. J. Environ. Res. Public Health.

[40] Hoffman M.D., White M.D. (2020). Belief in the need for sodium supplementation during ultramarathons remains strong: Findings from the Ultrarunners Longitudinal TRAcking (ULTRA) study. Appl. Physiol. Nutr. Metab..

[41] Krabak B.J., Lipman G.S., Waite B.L., Rundell S.D. (2017). Exercise-Associated Hyponatremia, Hypernatremia, and Hydration Status in Multistage Ultramarathons. Wilderness Environ. Med..

[42] Bonilla D.A., Pérez-Idárraga A., Odriozola-Martínez A., Kreider R.B. (2020). The 4R’s Framework of Nutritional Strategies for Post-Exercise Recovery: A Review with Emphasis on New Generation of Carbohydrates. Int. J. Environ. Res. Public Health.

[43] Valentin S., Pham L.A., Macrae E. (2021). Enablers and Barriers in Ultra-Running: A Comparison of Male and Female Ultra-Runners. Sport Soc..

[44] Partyka A., Waśkiewicz Z. (2024). Motivation of Marathon and Ultra-Marathon Runners: A Narrative Review. Psychol. Res. Behav. Manag..

[45] Zhao X., Chen Y., Li X., Wen W., Zhang J., Qiu J. (2025). Gastrointestinal Symptoms Among Recreational Long Distance Runners in China: Prevalence, Severity, and Contributing Factors. Front. Nutr..

[46] Ferrer D.A., Baumann C., Brandenberger K., Ellis R., Otis J. (2015). Physical Motivation Influences Race Performance over a 24-Hour Ultra-Marathon. Int. J. Sport Stud..

[47] Madaras L., Bowman J., Buckworth J. (2015). Motivational Differences Between Half, Full and Ultramarathoners. J. Sport Behav..

[48] Rozmiarek M., Malchrowicz-Mośko E., León-Guereño P., Tapia-Serrano M.Á., Kwiatkowski G. (2021). Motivational Differences Between 5K Runners, Marathoners and Ultramarathoners in Poland. Sustainability.

[49] Colangelo J., Smith A., Henninger K., Buadze A., Liebrenz M. (2025). Exploring the Presentation of REDs in Ultra-Endurance Sport: A Review. J. Eat. Disord..

[50] Ou L.B., Moriello C., Douros A., Filion K.B. (2021). Domperidone and the Risks of Sudden Cardiac Death and Ventricular Arrhythmia: A Systematic Review and Meta-Analysis of Observational Studies. Br. J. Clin. Pharmacol..

[51] Costa R.J.S., Snipe R., Camões-Costa V., Scheer V., Murray A. (2016). The Impact of Gastrointestinal Symptoms and Dermatological Injuries on Nutritional Intake and Hydration Status During Ultramarathon Events. Sports Med. Open.

[52] Williamson E. (2016). Nutritional Implications for Ultra-Endurance Walking and Running Events. Extreme Physiol. Med..

[53] Henninger K., Pritchett K., Brooke N.K., Dambacher L. (2024). Low Energy Availability, Disordered Eating, Exercise Dependence, and Fueling Strategies in Trail Runners. Int. J. Exerc. Sci..

[54] Ollitrault P., Milliez P., Alexandre J. (2023). Cardiovascular Toxicities Associated with Loperamide Use. Eur. Heart J. Case Rep..

[55] Ryan T., Daly E., Ryan L. (2023). Exploring the Nutrition Strategies Employed by Ultra-Endurance Athletes to Alleviate Exercise-Induced Gastrointestinal Symptoms—A Systematic Review. Nutrients.

[56] Atkins L., Francis J., Islam R., O’Connor D., Patey A., Ivers N., Foy R., Duncan E.M., Colquhoun H., Grimshaw J.M. (2017). A Guide to Using the Theoretical Domains Framework of Behaviour Change to Investigate Implementation Problems. Implement. Sci..

