# Supplementary material for: Understanding the Behavioural Determinants of Nutrition Practices in Ultra-Endurance Runners in Ireland
Source: Sports (Basel). 2026 Mar 11;14(3):109. doi: 10.3390/sports14030109 (PMC13030176; doi:10.3390/sports14030109)
Supplement: Supplementary file 1 [file sports-14-00109-s001.zip › sports-4134160-supplementary.pdf]

Table S1. Table outlining the International Society of Sports Nutrition Position Statement recommendations for energy, carbohydrates, protein, fats, liquids, and sodium intake during single-stage ultra-endurance running training and racing, and how to mitigate and manage gastrointestinal symptoms (GIS) [14].

	Recommendations for Training (% of Energy)	Recommendations for Racing
**Energy**	38-63kcal.kg.day	150-400kcal.hr for <50 mile events,<br><br>200-400kcal.hr for >50 mile events
**Carbohydrates**	7.0-10.0g.kg.day (60%)	30-50g.hr
**Protein**	1.6-2.1g.kg.day (15%)	5-10g.hr
**Fat**	1.0-1.5g.kg.day (25%)	1.1-1.7g.hr
**Hydration**	Not specified – drink to thirst	450-750ml.hr
**Sodium**	460mg.L post training, either in food or as a supplement	>575mg.L
**GIS Management**	Use strategies which aim to support GI adaptations that optimise CHO absorption	Avoid high concentrations of CHO,<br><br>Minimise dehydration,<br><br>Slow pace,<br><br>Reduce calories (but avoid persistent <200kcal.hr)

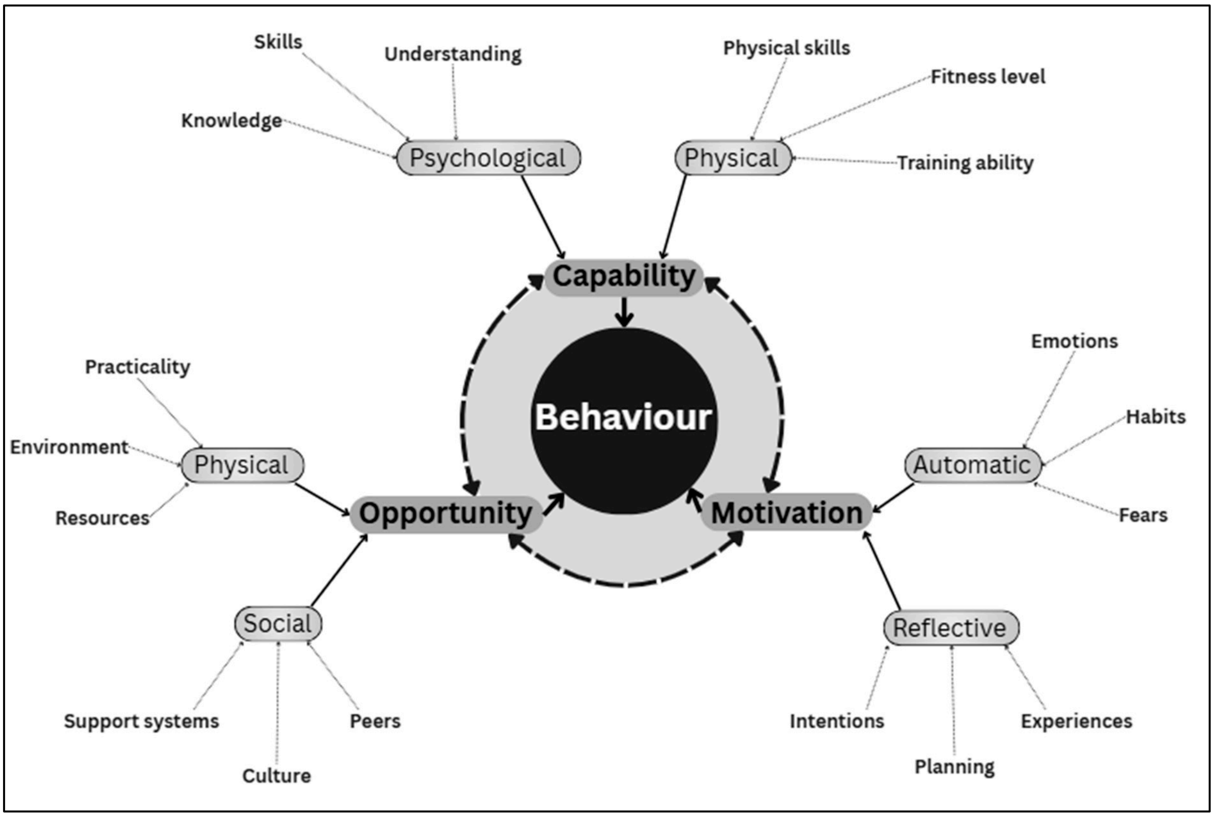

Figure S1. A depiction of the COM-B model, its components capability (C), opportunity (O), and motivation (M), and examples of topics which exist within each sub-component, adapted from Michie et al. [20].

Table S2. Examples of The Theoretical Domains Framework (TDF, version 2) domains, definitions, and their comprising constructs, adapted from "A guide to using the Theoretical Domains Framework of behaviour change to investigate implementation problems" by Atkins et al. [56].

Domain	Description	Constructs
Knowledge	Awareness of the existence of something	Knowledge (including knowledge of condition/scientific rationale)<br>Procedural knowledge<br>Knowledge of task environment
Skills	Abilities acquired through practice	Skills<br>Skills development<br>Competence<br>Ability<br>Interpersonal skills<br>Practice<br>Skill assessment
Beliefs about consequences	Recognition and acceptance of the real-world effects of actions within a given situation	Beliefs<br>Outcome expectancies<br>Characteristics of outcome expectancies<br>Anticipated regret<br>Consequents

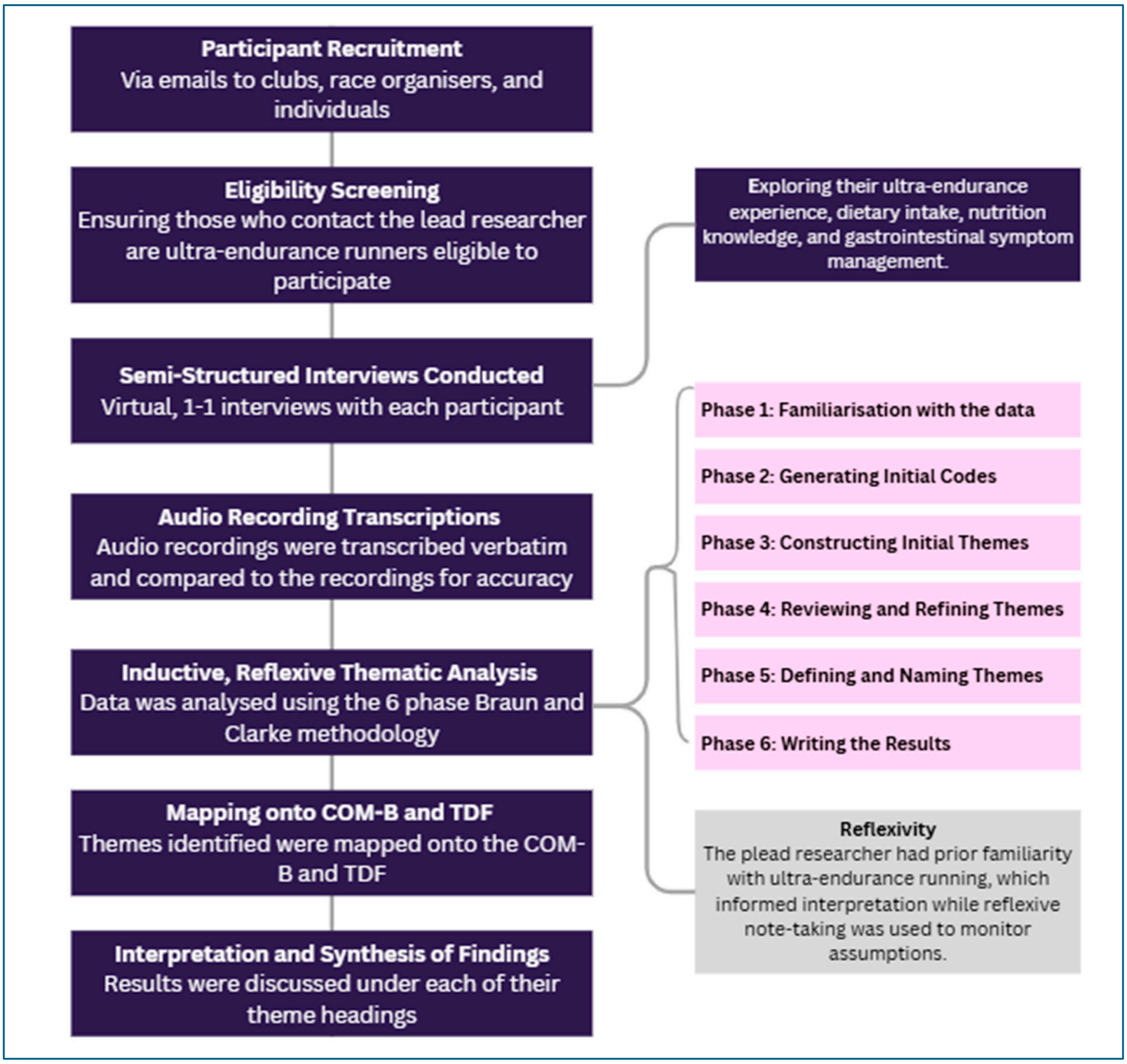

Figure S2. Illustration providing an overview of the research process and analytic steps undertaken, illustrating how data were generated, analysed, and interpreted within the COM-B and TDF frameworks.

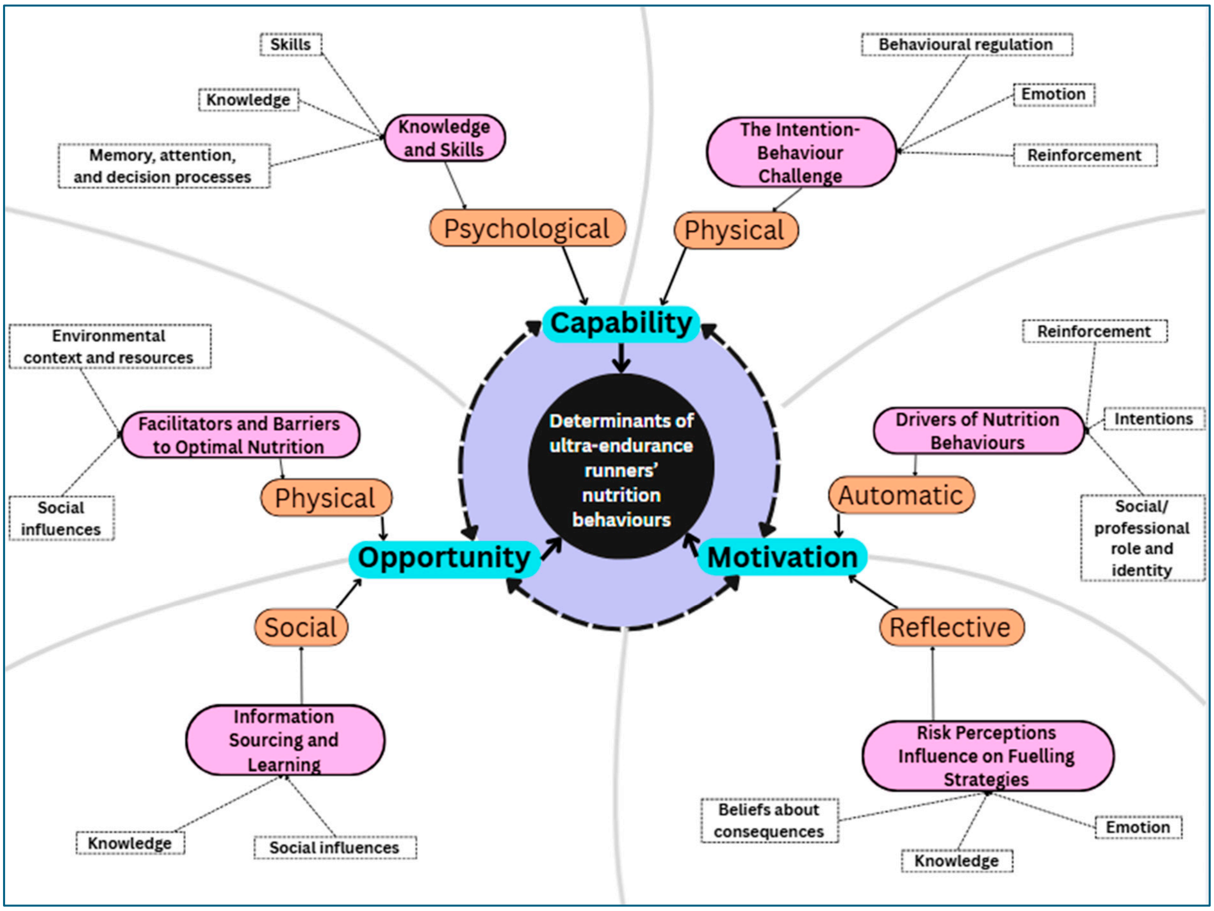

Figure S3. Behavioural determinants of nutrition practices in ultra-endurance runners who participated in the present study, mapped in accordance with the COM-B (capability, opportunity, and motivation) model of behaviour change and the Theoretical Domains Framework domains.

Table S3. Participants' running history (when they started running, their initial reasoning for running, if they always had an interest in pursuing ultra-endurance running, and weekly training volume), whether they employ strategies to manage gastrointestinal symptoms (GIS), their self-reported level of nutrition knowledge rated on a scale from 1, no knowledge, to 10, extremely knowledgeable, and their sources of nutrition information.

Participant number	Started running (age)	Initial Reason for running	Initial ultra intentions	Weekly training volume	GIS management strategies	Self-reported knowledge (/10)	Nutrition information sources
**1**	46	Hip injury rehabilitation	None states, but always wanted to do a marathon	90-110km	Reduced food volume,<br>Salty foods instead of salt,<br>No carbohydrate-loading,	6.5	Friends,<br>Other athletes,<br>Podcasts – particularly with qualified individuals,<br>Trial-and-errors
**2**	38	Doctors' advice after health scare	None stated	Not defined	Avoid specific foods	6/7	Other athletes,<br>Trial-and-error
**3**	29	Friends influence to begin trail running	None stated, but is self-proclaimed adventure lover	120-200km	Avoid specific foods	6/7	Friend who was nutritionist<br>Friends,<br>Trial-and-error,
**4**	22	Death of relative	None stated	50-60miles	Avoid specific foods	5/6	Google,<br>Social media,<br>Trial-and-error
**5**	26	Friends influence,<br>Death of family friend,<br>Life if short moment	Interested, went straight to ultra after short distances	40-80km	Avoid specific foods	6	Mother<br>Online,<br>Other athletes,<br>Trial-and-error,
**6**	31	Couch-to-5k,	None stated	<50 miles	Avoid specific foods	8	Books,<br>Running magazines,<br>Running websites,<br>Trial-and-error,

**7**	36	Influence of brother, keep fit during pandemic	Always interested	Up to 700k steps	Avoid specific foods	Not reported	Friends,<br>Partner
**8**	39	Previous policeman,<br>Keep fit, sparked by charity marathon	None stated	140k steps	Avoid specific foods	7	Online,<br>Other runners,<br>Running websites,<br>Trial-and-error
**9**	25 (“mid-20’s”)	Began as walking to stay active	None stated	100km +	Avoid specific foods	Not reported	Other athletes,
**10**	45	Retired from rowing career,<br>Keep fit	None stated, but likes chasing new goals	80-130km	Avoid lots of gels	8	Sport background,<br>Other professionals

Table S4. Example foods and beverages that participants often focus on including or avoiding before and during an ultra-endurance running event, whether, when asked, they reported experiencing GIS, whether in their interview they described having experienced GIS, and the supplements they include in their diet.

Participant number	Nutrition-related focus for an event	Example foods that participants often include before or during an event	Example foods that participants avoid before or during an event	Participants reported, when asked, whether they experience GIS	Participants described having previously experiencing GIS	Reported supplements used
**1**	Fast-releasing carbohydrates, Low food volume, Avoiding food close to the race start time	***Pre-event:*** Liquid carbohydrates, Fibre, ***During event:*** Jellies, Flapjacks, Biscuits, Fruits, Cola	Pasta, Meats, Potatoes, Rice, Chocolate, Crisps, Cheese, Chips	No	Yes	Salt
**2**	Hydration	***Pre-event:*** Vegetarian food, Porridge, Pizza, Pasta, Bread, ***During event:*** Bananas, Confectionary (jellies, chocolate), Fruit, Rice pudding	Greens, Fibre, Cola, Sports drinks, Caffeine gels	No	Yes	Casein, Gels, Caffeine, Loperamide
**3**	None	***Pre-event:*** Fruit, ***During event:*** None	Avoids all food	No	Yes	None
**4**	Hydration	***Pre-event:*** Pasta, Rice, Meat with every dinner, ***During event:*** Flapjacks, Bars, Cheese sandwiches, Peanut butter sandwiches, Boiled potatoes, Pretzels, Crisps	Biscuits, Sweets, Alcohol	Yes	Yes	Hydration tablets/ Salt, Gels

**5**	Hydration, Carbohydrates	***Pre-event:*** Pizza, Bread, Hummus, Rice, ***During event:*** Rice cakes, Bars, Pot noodles, Flat cola,	Coffee, Multi-ingredient shakes/drinks, Red meat	Small amounts	Yes	Electrolytes, Gels, Domperidone, Many vitamins
**6**	Hydration, Carbohydrates	***During event:*** Sweets, Flat sports drinks, Oat bars, Jellies, Crackers, Biscuits, Fig rolls, Coconut water	Spicy food, Fibre, Egg sandwiches, Sausages, Pizza, Chocolate, Curry, high fat foods	Small amounts	Yes	Electrolytes, Omegas, Green blend, Salt,
**7**	Salt, Fast-releasing carbohydrates	***During event:*** Cola, High sugar foods, chocolate	None specified	No	Yes	Salt, Energy gels
**8**	Hydration	***During event:*** Energy bars, Bananas, Flat cola, Nutrition bars, Rice pudding	Sports drinks, Spicy food, Meats (particularly red), Desserts, High sugar foods, Chocolate	Sometimes	Yes	Salt, Gels, Multivitamin sachets
**9**	Carbohydrates, Hydration Protein (in recovery period)	***During event:*** Jellies, Porridge	Cheese	Not usually	Yes	Magnesium, Salt, Energy Gels
**10**	Hydration, Salt, Protein (in recovery period)	***During event:*** Bars, Flapjacks, Beef jerky, Sweets, Bananas,	High consumption of sports gels or bars	No	Rarely, self-reported "iron stomach"	Salt, Caffeinated gels and Decaffeinated gels, Recovery shake
